# Integrated Analysis of Bulk RNA-Seq and Single-Cell RNA-Seq Unravels the Influences of SARS-CoV-2 Infections to Cancer Patients

**DOI:** 10.3390/ijms232415698

**Published:** 2022-12-10

**Authors:** Yu Chen, Yujia Qin, Yuanyuan Fu, Zitong Gao, Youping Deng

**Affiliations:** 1 Department of Quantitative Health Sciences, John A. Burns School of Medicine, University of Hawaii at Manoa, Honolulu, HI 96813, USA; 2 Department of Molecular Biosciences and Bioengineering, College of Tropical Agriculture and Human Resources, University of Hawaii at Manoa, Honolulu, HI 96822, USA

**Keywords:** SARS-CoV-2, cancer, pulmonary fibrosis, acute respiratory distress, PPI, drug molecule, single-cell RNA-seq, immunity, monocyte, m6A

## Abstract

Severe acute respiratory syndrome coronavirus 2 (SARS-CoV-2) is a highly contagious and pathogenic coronavirus that emerged in late 2019 and caused a pandemic of respiratory illness termed as coronavirus disease 2019 (COVID-19). Cancer patients are more susceptible to SARS-CoV-2 infection. The treatment of cancer patients infected with SARS-CoV-2 is more complicated, and the patients are at risk of poor prognosis compared to other populations. Patients infected with SARS-CoV-2 are prone to rapid development of acute respiratory distress syndrome (ARDS) of which pulmonary fibrosis (PF) is considered a sequelae. Both ARDS and PF are factors that contribute to poor prognosis in COVID-19 patients. However, the molecular mechanisms among COVID-19, ARDS and PF in COVID-19 patients with cancer are not well-understood. In this study, the common differentially expressed genes (DEGs) between COVID-19 patients with and without cancer were identified. Based on the common DEGs, a series of analyses were performed, including Gene Ontology (GO) and pathway analysis, protein–protein interaction (PPI) network construction and hub gene extraction, transcription factor (TF)–DEG regulatory network construction, TF–DEG–miRNA coregulatory network construction and drug molecule identification. The candidate drug molecules (e.g., Tamibarotene CTD 00002527) obtained by this study might be helpful for effective therapeutic targets in COVID-19 patients with cancer. In addition, the common DEGs among ARDS, PF and COVID-19 patients with and without cancer are TNFSF10 and IFITM2. These two genes may serve as potential therapeutic targets in the treatment of COVID-19 patients with cancer. Changes in the expression levels of TNFSF10 and IFITM2 in CD14+/CD16+ monocytes may affect the immune response of COVID-19 patients. Specifically, changes in the expression level of TNFSF10 in monocytes can be considered as an immune signature in COVID-19 patients with hematologic cancer. Targeting N^6^-methyladenosine (m6A) pathways (e.g., METTL3/SERPINA1 axis) to restrict SARS-CoV-2 reproduction has therapeutic potential for COVID-19 patients.

## 1. Introduction

At the end of 2019, an acute respiratory disease called coronavirus disease 2019 (COVID-19) was identified in China and then spread and became a worldwide pandemic, threatening human health and public safety. The disease is caused by infection with a virus named Severe acute respiratory syndrome coronavirus 2 (SARS-CoV-2), a novel coronavirus that is highly contagious and pathogenic [1].

Cancer patients are more susceptible to viral infections due to a range of factors, such as the poor health status, the presence of other chronic diseases and their systemic immunosuppression states caused by cancer and anticancer therapy. Therefore, the treatment of cancer patients infected with SARS-CoV-2 is more complicated, and they are at risk of poor prognosis compared to other populations [2]. Based on the large cohort study by Dai et al. (2020), which included COVID-19 patients with cancer (*n* = 105) and COVID-19 patients without cancer (*n* = 536), COVID-19 patients with cancer were at higher risk for all serious outcomes compared to COVID-19 patients without cancer [3].

Even for COVID-19 patients without cancer, there are patients who have been detected with high viral loads and develop severe respiratory symptoms. These patients often experience dyspnea and hypoxemia and rapidly develop acute respiratory distress syndrome (ARDS) [4]. ARDS is a clinical manifestation of acute lung injury. The severity of ARDS is closely related to the patient mortality rate. In a retrospective cohort study by Wu et al. (2020), among a total of 201 COVID-19 patients, 84 patients developed ARDS symptoms, and 67 received mechanical ventilation. All patients who died developed ARDS and received mechanical ventilation [5]. In addition, based on a clinical study of 5700 COVID-19 patients in the New York City area, patients who developed ARDS and required mechanical ventilation had a mortality rate of 88.1%. The results of these studies highlighted the fact that the development of ARDS was a leading cause of death in patients infected with SARS-CoV-2 [6].

Pulmonary fibrosis (PF) is considered a sequelae of ARDS [7]. In a study of pulmonary function from 110 patients who recovered from COVID-19 at time of hospital discharge, more than one-third of the recovered patients developed abnormal pulmonary fibrosis. In addition, 47.2% of recovered patients had impaired lung diffusing capacity for carbon monoxide, and 25% of recovered patients had decreased total lung capacity [8]. Notably, progressive pulmonary fibrosis has been shown to be the leading cause of death in the majority of ARDS patients [7]. A significant proportion of survivors have radiographic abnormalities due to the presence of pulmonary fibrosis [9], and they also experienced long-term damage to lung functions [10]. The burden of post-COVID-19 pulmonary fibrosis can be substantial for a subset of patients as they age [11]. Just a relatively small degree of residual, not even progressive fibrosis, can contribute to considerable morbidity and mortality in the elderly people infected with SARS-CoV-2, especially when many of them may already have lung diseases.

**Figure 1:**
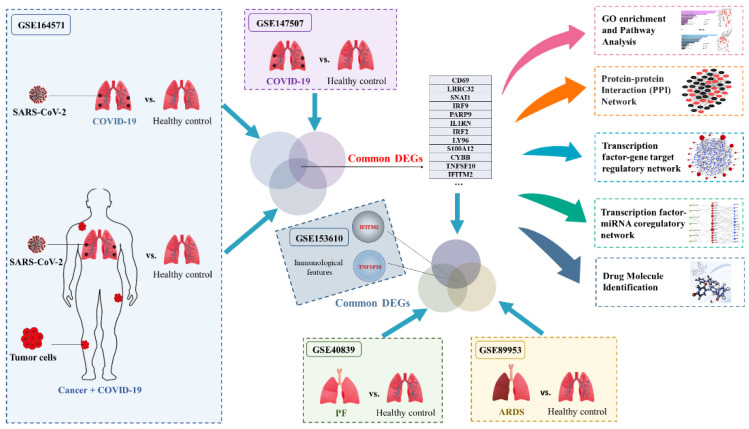
The workflow of current analysis. DEGs were identified between COVID-19 patients without cancer and healthy controls by using the GSE164571 and GSE147507 datasets. Then we identified the common DEGs between COVID-19 patients with and without cancer. Based on the common DEGs, a series of analyses were performed, including GO and pathway analysis, PPI network construction and hub gene extraction, transcription factor–DEG regulatory network construction, transcription factor–DEG–miRNA coregulatory network construction and drug molecule identification. In addition, the common DEGs among ARDS, PF and COVID-19 patients with and without cancer were TNFSF10 and IFITM2. The immunological features of potential biomarkers were also validated in COVID-19 patients with hematological cancer using single-cell RNA-seq analysis based on GSE153610.

In this study, several Gene Expression Omnibus (GEO) datasets were used to explore biological relationships and potential therapeutic targets among COVID-19 patients with and without cancer, and how these targets were related to ARDS and PF. Differentially expressed genes (DEGs) were identified between COVID-19 patients and healthy controls by using the GSE164571 and GSE147507 datasets. Then we identified the common DEGs between COVID-19 patients with and without cancer, comparing them to the DEGs identified for COVID-19 patients earlier. These DEGs were used for the downstream analysis to explore the systematic changes caused by COVID-19 and cancers based on the transcriptomics patterns. First, Gene Ontology (GO) and pathways analysis based on these common DEGs were performed to elucidate the biological processes of genome-based expression studies. The protein–protein interaction (PPI) network was constructed from the common DEGs to reveal the most significant connections between these proteins of interest, while the hub genes were also identified from the PPI network. The construction of transcription factor (TF)–DEG interactions and miRNA–DEG–TF coregulatory network elucidated the ways in which DEG were regulated at the transcriptional and post-transcriptional levels. Based on the principle of protein–drug interactions, drug molecules with potential value for the treatment of COVID-19 patients with cancer were predicted. In addition, two genes were identified as the common DEGs among ARDS, PF and COVID-19 cancer patients: TNFSF10 and IFITM2. These two genes may serve as potential therapeutic targets in the treatment of COVID-19 patients with cancer. The immunological features of potential biomarkers were also explored and validated in other COVID-19 cohorts using single-cell RNA-seq analysis based on the GSE153610 dataset. The workflow of the current analysis is displayed in Figure 1.

## 2. Results

### 2.1. Transcriptomic Differences between SARS-CoV-2 and Healthy Control

**Figure 2:**
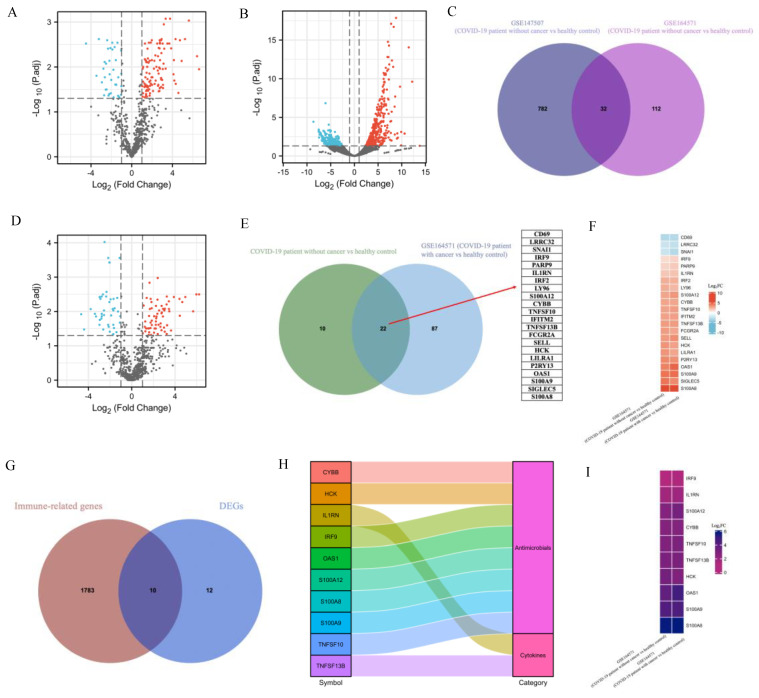
Identification of DEGs and screening of common DEGs between COVID-19 patients with and without cancer. The volcano plot depicts the DEGs between COVID-19 patients without cancer and healthy controls by using the GSE164571 (**A**) and GSE147507 (**B**) datasets (Adjusted *p*-value < 0.05, |log2FC| ≥ 1). Based on DEGs in (**A**,**B**), the common DEGs were shown through a Venn diagram (**C**). Identification of DEGs between COVID-19 patients with cancer and healthy control by using the GSE164571 dataset (**D**) (Adjusted *p*-value < 0.05, |log2FC| ≥ 1). The Venn diagram (**E**) shows the common DEGs between 32 common genes derived from (**C**) and DEGs derived from (**D**). (**F**) The heatmap shows gene expression changes in the following two groups of the common DEGs from (**E**) in the GSE164571 dataset: COVID-19 patient without cancer vs healthy control, COVID-19 patient with cancer vs healthy control. (**G**) The Venn diagram shows the common genes between 22 DEGs obtained from Figure 2E and immune-related genes. (**H**) The 10 immune-related genes obtained from Figure 2G were classified and visualized by a Sankey diagram. (**I**) Based on the GSE164571 dataset, the expression levels of 10 immune-related genes obtained from Figure 2G were visualized in different groupings by heatmap.

The GSE164571 dataset was used for the DEGs identification between COVID-19 patients with and without cancer [12]. Twelve peripheral blood mononuclear cell (PBMC) samples were divided into three groups: two healthy controls, five COVID-19 patients without cancer and five COVID-19 patients with cancer. We first identified DEGs between COVID-19 patients without cancer and healthy controls. Among 784 genes were analyzed, 144 DEGs were identified as significant, including 107 upregulated genes and 37 downregulated genes (Figure 2A). We then used the GSE147507 dataset to narrow down the list of DEGs [13], where 9510 genes were obtained from the RNA-seq data. Compared with uninfected human lung samples, there were 814 DEGs, including 395 upregulated and 419 downregulated genes from human lung samples, infected with SARS-CoV-2 (Figure 2B). The comparison of the common DEGs between SARS-CoV-2 and healthy control from the above two GEO datasets was plotted using a Venn diagram (Figure 2C), where 32 common DEGs were finally identified.

### 2.2. Identification of DEGs and Screening of Common Genes between COVID-19 Patients with and without Cancer

Based on data from the GSE164571 dataset, comparing COVID-19 patients with cancer and healthy control, we identified 66 upregulated and 43 downregulated genes from 109 DEGs (Figure 2D). These 109 DEGs were compared with the 32 DEGs obtained in Figure 2C, and 22 common DEGs (Figure 2E) were found to be the overlapped genes that had different expression patterns comparing COVID-19 vs. healthy control and comparing COVID-19 patients with and without cancers. For example, it is shown in Figure 2F that, in the GSE164571 dataset, compared with healthy control, CD69, LRRC32 and SNAI1 were downregulated in COVID-19 patients with or without cancer, while 19 other genes were upregulated in COVID-19 patients with or without cancer.

We further explored whether these 22 DEGs were involved in the immune system, and in Figure 2G, it is shown that, among the 22 DEGs, 10 genes were immune-related genes. In Figure 2H, the 10 immune-related genes were classified into two categories: antimicrobials (CYBB, HCK, IRF9, OAS1, S100A12, S100A8, S100A9, TNFSF10) and cytokines (IL1RN, TNFSF13B). In Figure 2I, it is highlighted that, in the GSE164571 dataset, compared with healthy control, the 10 immune-related genes were upregulated in COVID-19 patients with or without cancer. Therefore, it suggested that the corresponding immune responses were activated in COVID-19 patients with cancer or not.

### 2.3. Common Genes-Based GO and Pathway Analysis

**Figure 3:**
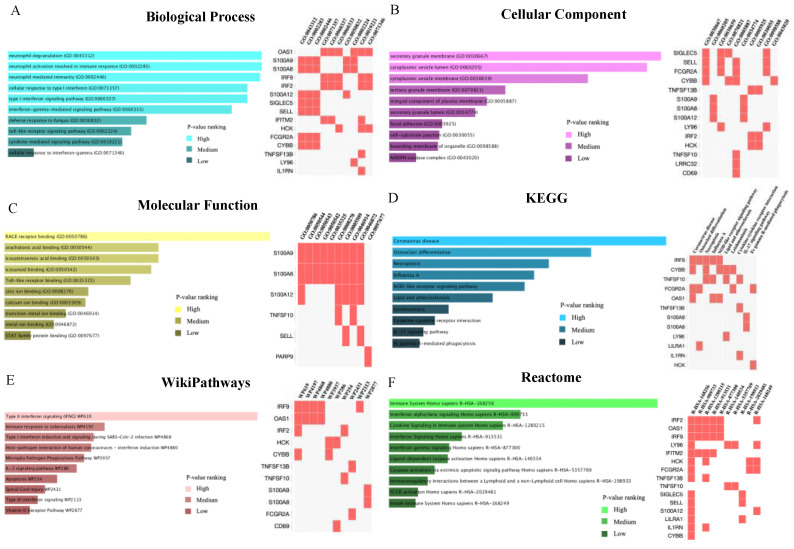
Common genes-based GO and pathway analysis sorted by adjusted *p*-values. Enriched genes are marked. (**A**–**C**) GO terms analysis results were shown, including biological process, cellular component and molecular function. (**D**–**F**) Pathway analysis results were identified by KEGG (Kyoto Encyclopedia of Genes and Genomes), WikiPathways and Reactome.

The following 22 common DEGs (identified earlier) were used to performed gene enrichment analysis via Enrichr platform: CD69, LRRC32, SNAI1, IRF9, PARP9, IL1RN, IRF2, LY96, S100A12, CYBB, TNFSF10, IFITM2, TNFSF13B, FCGR2A, SELL, HCK, LILRA1, P2RY13, OAS1, S100A9, SIGLEC5 and S100A8. The combined score performed by the Enrichr platform was calculated by the logarithm of the adjusted *p*-value. Genes that were enriched in the top 10 GO terms and pathways were listed in Figure 3. The results of the GO analysis were summarized into three categories: biological process, cellular component and molecular function. For the biological process category, the common DEGs were highly enhanced in neutrophil-mediated immune responses and interferon-related pathways. The result of the cellular component analysis shows that secretory granule membrane, cytoplasmic vesicle lumen and cytoplasmic vesicle membrane are significantly involved by the common DEGs, while the most significant changed molecular function was RAGE (receptor for advanced glycation end products) receptor binding. The most impacted pathways involved by these common DEGs between COVID-19 patients with and without cancer were identified based on three databases: KEGG (Kyoto Encyclopedia of Genes and Genomes), WikiPathways and Reactome. Type II interferon signaling pathway, related pathways of coronavirus disease (COVID-19) and immune response are the top pathways enriched by the common DEGs. Four genes: FCGR2A (FcRII–A), IRF9, OAS1 (2′–5′-oligoadenylate synthetase 1) and CYBB (also known as NOX2) were highlighted in the related pathways of coronavirus disease (COVID-19) from KEGG database (Figure S1).

### 2.4. PPI Network Construction and Hub Genes Extraction

**Figure 4:**
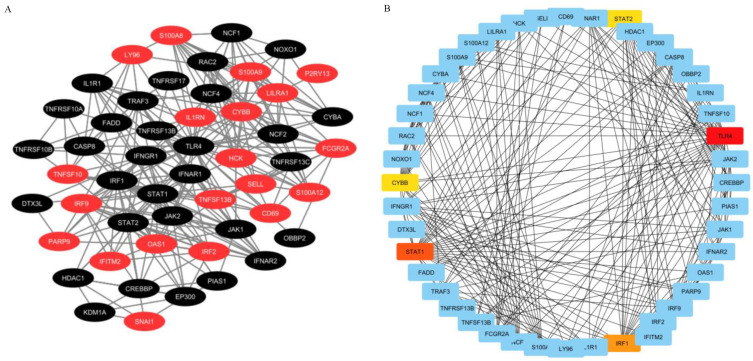
PPI network construction and hub genes extraction. (**A**) Protein–protein interactions (PPI) network of common DEGs between COVID-19 patients with and without cancer. The common genes are highlighted with red nodes. The network contains 51 nodes and 542 edges. (**B**) The hub genes were extracted from the PPI network of common DEGs. According to the degree value, the highlighted TOP5 hub genes are TLR4, STAT1, IRF1, CYBB and STAT2. The network is made up of 47 nodes and 263 edges.

The 22 common DEGs were provided as input in STRING database, and the generated file was then imported into Cytoscape software for visualization of the PPI network. The PPI network can help to further analyze the interactions among the common DEGs obtained in this study, such as extraction of the hub genes related to them. The highlighted common DEGs in the PPI network were marked in red (Figure 4A), and the network consisted of 51 nodes and 542 edges. In a PPI network, the most interconnected nodes are considered as hub genes. In the Cytoscape software, the cytoHubba plugin was used to identify the hub genes in the PPI network by the degree algorithm (Figure 4B). Sorted by the degree value of each gene, the top five hub genes were identified as: TLR4, STAT1, IRF1, STAT2 and CYBB (NOX2). These hub genes can be regarded as potential biomarkers and may also bring new perspectives for the treatment of diseases.

### 2.5. TF–DEG Interactions and miRNA–DEG–TF Coregulatory Networks

**Figure 5:**
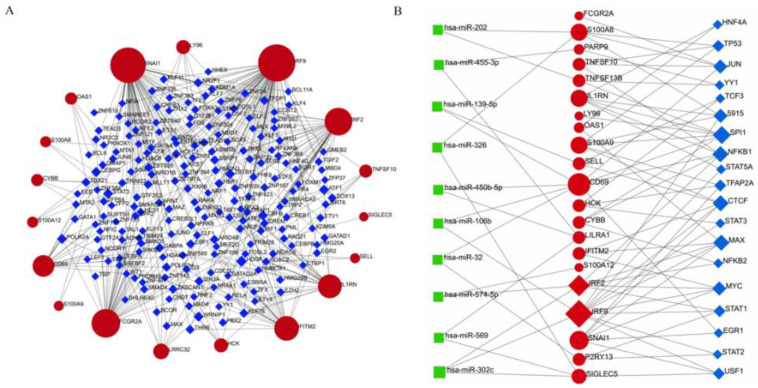
The construction of TF–DEG and TF–DEG–miRNA co-regulatory networks. (**A**) TF–DEG interactions network visualization. The network contained 215 nodes and 325 edges. Common DEGs were marked in red. TF genes were marked in blue. (**B**) TF–DEG–miRNA co-regulatory network identification. The network included 50 nodes and 82 edges. There were 10 miRNAs (green) and 19 TF genes (blue) in the network, and they interacted with common DEGs (red).

TF and miRNA interactions with DEGs can be considered as an important way to examine the DEGs from expression changes at the transcriptional and post-transcriptional levels. TFs–DEGs regulatory network was generated by the NetworkAnalyst platform. The common DEGs interaction with TF-genes was depicted in Figure 5A. The network contained 215 nodes and 325 edges, and 197 TF regulators were included in the network. Notedly, IRF9 was regulated by 77 TF-genes and SNAI was regulated by 67 TF-genes. The common DEGs were regulated by one or even multiple TF-genes, suggesting that DEGs were highly interacting with different TF-genes. In the miRNA–DEG–TF coregulatory network (Figure 5B), DEG expressions can be regulated not only by TF-genes, but also by more than one post-transcriptional regulatory signature (miRNAs). The network contained 50 nodes and 82 edges, among which there were 10 miRNAs and 19 TF-genes. Only TF-genes or miRNAs that regulated more than one DEGs were shown in the figure.

### 2.6. Prediction of Drug Candidate Molecules

**Figure 6:**
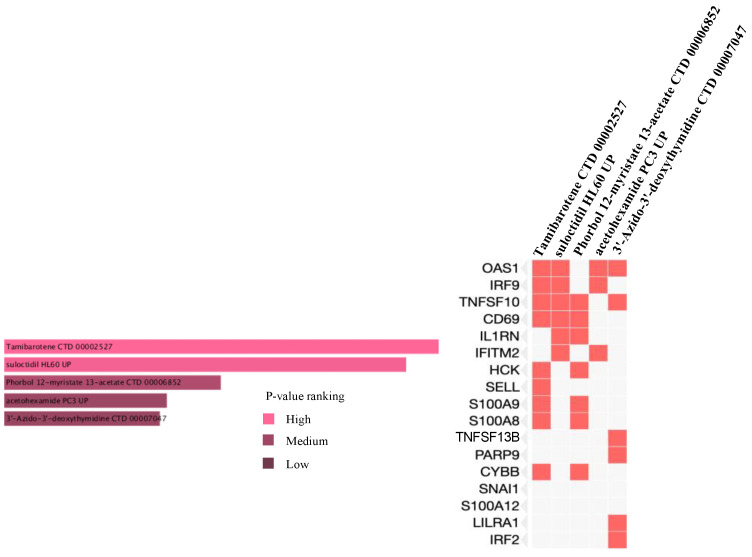
Prediction of drug candidate molecules. The drug candidate molecules were predicted by the DSigDB database of the Enrichr web platform. The results were based on the interaction of 22 common genes with drugs and sorted by adjusted P-values. Enriched genes were marked.

The drug candidate molecules were predicted by the Drug Signatures Database (DSigDB) of the Enrichr web platform. The results were based on the interaction of 22 common genes with drugs molecules. According to adjusted p-value, Figure 6 suggested the top 5 candidate drug molecules for COVID-19 patients with cancer. The results indicated that Tamibarotene CTD 00002527 and suloctidil HL60 UP were the two potential drug molecules that could interact with most genes. In Table 1, the information is summarized for the top 5 drug candidates in terms of chemical formula, chemical structures and background.

### 2.7. Identification of DEGs for ARDS Patients

**Figure 7:**
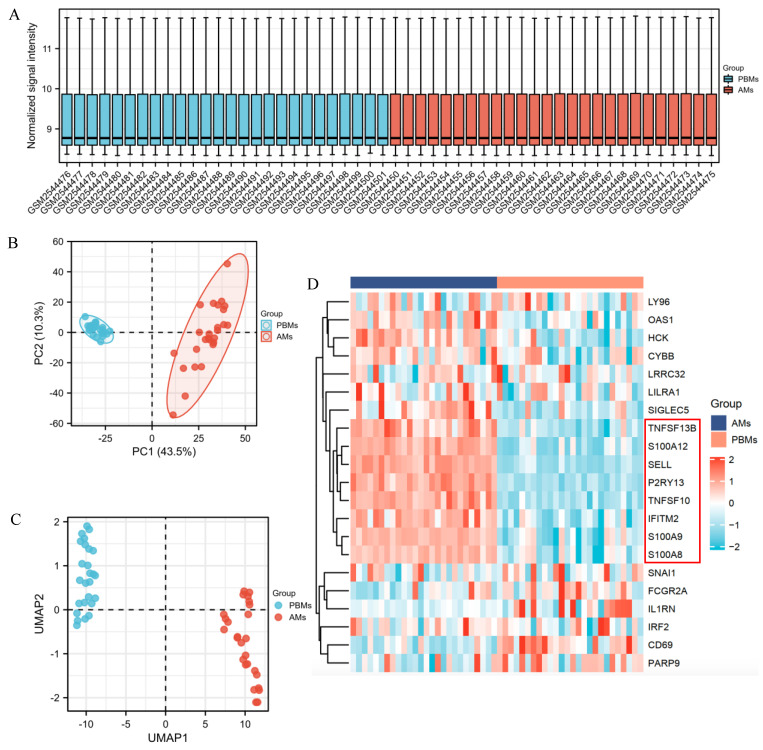
Identification of common DEGs between paired alveolar macrophages (AM) and peripheral blood monocyte (PBM) samples in acute respiratory distress syndrome (ARDS) patients. (**A**) The data were normalized. (**B**,**C**) PCA and UMAP plots indicated that the different categories (AM and PBM samples) were well differentiated. (**D**) The common DEGs obtained from Figure 2E were selected to make the heatmap. The expression data for IRF9 was missing and therefore not shown.

In general, infection and injury to the lungs is the main route by which SARS-CoV-2 infects patients, resulting in pneumonia, and a more serious condition, ARDS [23]. Therefore, the identification of DEGs in the pathogenesis of ARDS is crucial for the guidance of its treatment. Morrell et al. (2018) assessed transcriptional activation based on paired alveolar macrophages (AM) and peripheral blood monocytes (PBM) samples in human ARDS. The findings suggested that serial measurements of genome-wide transcriptional changes in AM and PBM from ARDS patients could clarify the biologic programs activated in ARDS. Moreover, the relationship between the transcriptional changes of the two was also closely related to clinical outcomes [24]. The GSE89953 dataset was used for the DEGs identification between PBMs and AMs in ARDS patients. The RNA-seq data from 26 paired AMs and PBMs samples were normalized (Figure 7A) and the PCA plot showed that transcriptome differences between different types of samples were quite obvious (Figure 7B). UMAP plot also indicated that different conditions can be well separated (Figure 7C). The expression of the common DEGs obtained from Figure 2E in different samples was shown in Figure 7D. The result showed that TNFSF13B, S100A12, SELL, P2RY13, TNFSF10, IFITM2, S100A9 and S100A8 were upregulated in AMs samples, while these 8 genes were downregulated in PBMs samples.

### 2.8. Identification of DEGs between PF and Healthy Control

**Figure 8:**
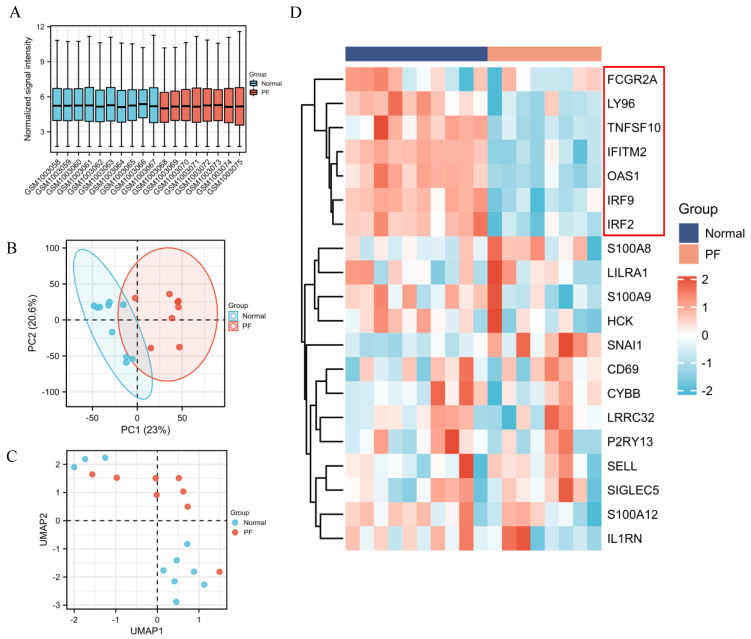
Identification of common DEGs between pulmonary fibrosis (PF) and healthy control. (**A**) The data were normalized. (**B**,**C**) PCA and UMAP plots indicated that the different categories (PF and normal samples) were well-differentiated. (**D**) The common DEGs obtained from Figure 2E were selected to make the heatmap. The expression data for TNFSF13B and PARP9 was missing and therefore not shown.

Many COVID-19 patients with critical symptoms may develop long-term lung damage after virus clearance, especially fibrotic interstitial lung disease. PF is a well-recognized sequela of ARDS [7]. We identified the DEGs between normal and fibrotic human lung tissues based on the GSE40839 dataset. The expression data from 10 normal and 8 fibrotic human lung tissue samples were normalized (Figure 8A), and the PCA plot and UMAP plot depicted that the different transcriptomes can be well differentiated (Figure 8B,C). The expression of common DEGs obtained from Figure 2E in different samples was shown in Figure 8D, and the heatmap showed that FCGR2A, LY96, TNFSF10, IFITM2, OAS1, IRF9 and IRF2 were downregulated in PF samples, while these 7 genes were upregulated in normal samples.

### 2.9. Identification of Common DEGs among COVID-19 Patients without and with Cancer, ARDS and PF

**Figure 9:**
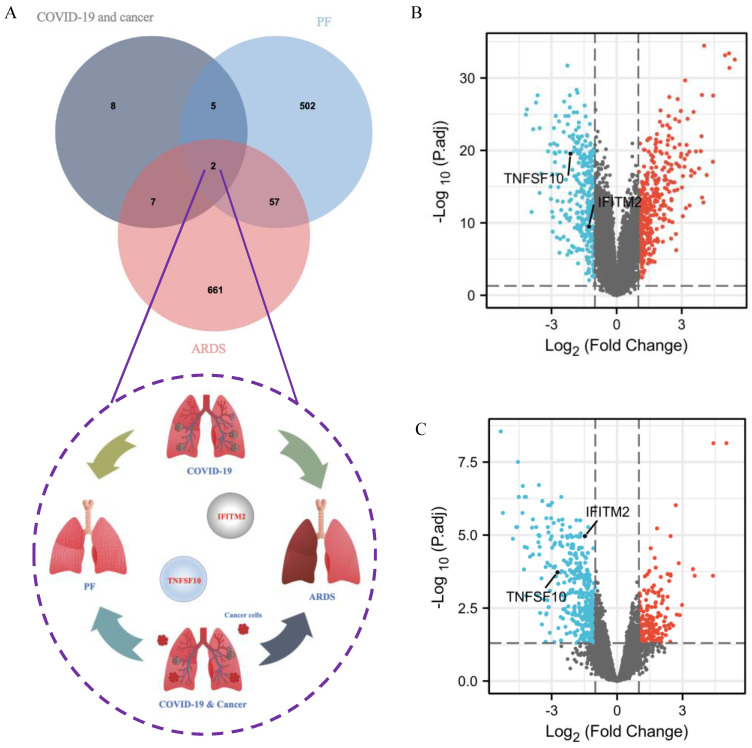
Identification of common DEGs among COVID-19 patients without and with cancer, ARDS and PF. (**A**) The Venn diagram suggested that TNFSF10 and IFITM2 are two very significant DEGs based on GSE164571, GSE147507, GSE40839 and GSE89953 datasets. TNFSF10 and IFITM2 were marked in the volcano plots from the GSE89953 dataset (ARDS) (**B**) and the GSE40839 dataset (PF) (**C**) (Adjusted *p*-value < 0.05, |log2FC| ≥ 1).

Given that ARDS and PF may be potentially adverse symptoms for subsequent treatment of cancer patients infected with SARS-CoV-2, the common DEGs were identified from SARS-CoV-2, ARDS and PF datasets. The result suggested that TNFSF10 and IFITM2 were the two most significant DEGs, which can be shown in the Venn diagram (Figure 9A). In Figure 2F, the heatmap showed that, in the GSE164571 dataset, compared with healthy control, TNFSF10 and IFITM2 were upregulated in COVID-19 patients with or without cancer. In the volcano plots, 404 upregulated and 323 downregulated genes were included in the obtained 727 DEGs from the ARDS dataset. TNFSF10 and IFITM2 were downregulated (Figure 9B). The 566 DEGs contained 169 upregulated and 397 downregulated genes based on the PF dataset. TNFSF10 and IFITM2 were downregulated (Figure 9C).

### 2.10. Single-Cell RNA-Seq Analysis Revealed Immunological Features of TNFSF10 and IFITM2 in COVID-19 Patients

**Figure 10:**
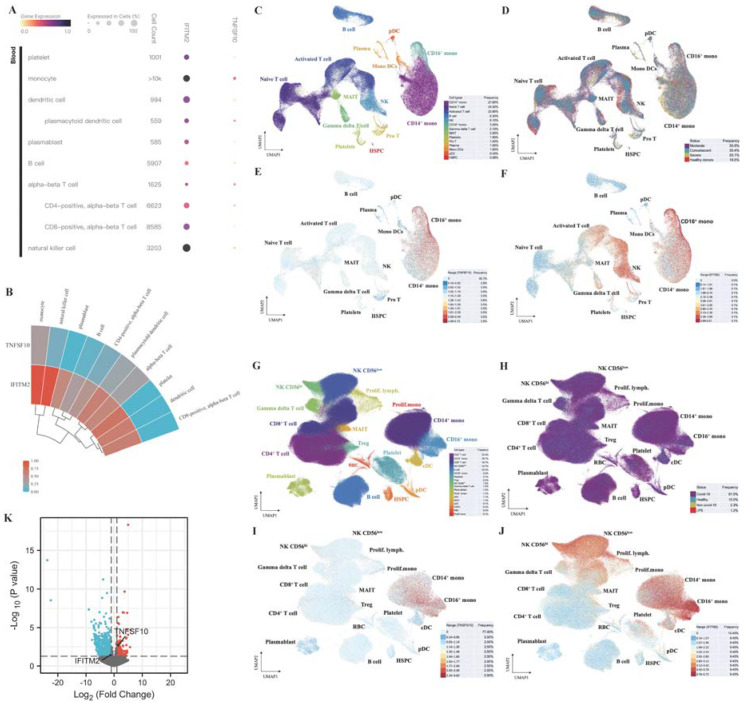
Immunological features of TNFSF10 and IFITM2 in COVID-19 patients with or without cancer by Single-cell RNA-seq analysis. (**A**,**B**) Based on the PBMC samples from the cellxgene dataset, the expression level of TNFSF10 and IFITM2 in COVID-19 patients without cancer was obtained by Single-cell RNA-seq analysis. More results were based on the single-cell RNA-seq data included in the publication of Zhang et al. (2021) [25] (**C**–**F**) and Stephenson et al. (2021) [26] (**G**–**J**). (**C**) The UMAP projection of 122,542 single cells from healthy donors (*n* = 5), moderate (*n* = 7), severe (*n* = 4) and convalescent (*n* = 6) COVID-19 patient samples. Each dot represents a single cell. The 14 clusters were marked with corresponding colors. (**D**) The distribution of PBMC cell samples among 14 clusters based on different clinical status. (**E**,**F**) The expression levels of TNFSF10 and IFITM2 in 14 clusters, respectively. (**G**) The UMAP projection of 781,123 single cells from 41 controls and 102 COVID-19 patients. Each dot represents a single cell. The 18 clusters were marked with corresponding colors. (**H**) The distribution of PBMC cells samples among 18 clusters based on different clinical status. (**I**,**J**) The expression levels of TNFSF10 and IFITM2 in 18 clusters, respectively. Different categories were distinguished by different colors, and the corresponding frequencies were marked. Lymph, lymphocyte; mono, monocyte; prolif, proliferating. (**K**) Compared with recovered COVID-19 patients without cancer, the volcano plot indicated changes in the expression levels of TNFSF10 and IFITM2 in monocytes of COVID-19 patients with hematologic cancer based on the GSE153610 dataset.

Based on the PBMC samples from the cellxgene dataset, the expression data of TNFSF10 and IFITM2 in COVID-19 patients were obtained by Single-cell RNA-seq analysis. The expression levels of IFITM2 and TNFSF10 in various immune cells were shown in Figure 10A. This heatmap indicated that both genes were highly expressed in monocytes. In addition, IFITM2 was also highly expressed in natural-killer (NK) cells (Figure 10B).

The single-cell RNA-seq data from Zhang et al. (2020) [25] were used to explore the immunological features of potential biomarkers TNFSF10 and IFITM2 in COVID-19 cohorts. The PBMC samples from 5 healthy donors and 13 COVID-19 patients were collected. Figure 10C shows that 122,542 single cells were divided into 14 clusters. These samples in different COVID-19 conditions were evenly distributed in the 14 clusters (Figure 10D). In this COVID-19 cohort, TNFSF10 was highly expressed in CD14+ and CD16+ monocytes (Figure 10E). IFITM2 was highly expressed in CD14+, CD16+ monocytes and NK cells (Figure 10F). We also used the single-cell RNA-seq data published by Stephenson et al. (2021) [26] to verify the above conclusions. PBMC samples from 41 controls and 102 COVID-19 patients were obtained. 781,123 single cells were classified into 18 clusters (Figure 10G,H). Figure 10I,J described similar conclusions: compared with other immune cells, TNFSF10 was highly expressed in CD14+ and CD16+ monocytes. IFITM2 was highly expressed in CD14+, CD16+ monocytes and NK cells.

The immunological features of potential biomarkers were also validated in COVID-19 patients with hematologic cancer using single-cell RNA-seq analysis. Based on the GSE153610 dataset (Figure 10K and Table S1), compared with recovered COVID-19 patients without cancer, TNFSF10 were upregulated in monocytes of cancer patients infected with SARS-CoV-2, while IFITM2 were downregulated.

## 3. Discussion

Given the susceptibility of cancer patients to SARS-CoV-2 infection, cancer patients are at high risk of developing respiratory complications associated with SARS-CoV-2 infection [27], and that is why cancer patients should require special care or treatment during the SARS-CoV-2 pandemic. Here, we were trying to search for genes that can provide some insights of the molecular mechanisms underlying the effects of SARS-CoV-2 infection in cancer patients.

In this study, GSE164571 and GSE147507 datasets were used to identify the common DEGs between COVID-19 patients without and with cancer. In Figure 2F, the heatmap showed that the 22 common DEGs were clearly distinguished in two different groups according to the changes of their expression levels. Importantly, 10 immune-related genes from 22 DEGs were upregulated in COVID-19 patients with or without cancer (Figure 2I). Previous studies have shown that innate and adaptive immune responses were activated after SARS-CoV-2 infection [28]. Our findings confirmed that the immune responses were activated in COVID-19 patients with or without cancer.

According to the results of KEGG (Figure 3) pathway analysis, related pathways of coronavirus disease (COVID-19) were identified based on the 22 common DEGs, which confirmed that these DEGs were involved in the response to the viral infection and makes the subsequent analysis based on these 22 common DEGs more convincing. The following four genes were enriched in the related pathways of coronavirus disease (COVID-19) from KEGG database: FCGR2A (FcRII–A), IRF9, OAS1(2′-5′-oligoadenylate synthetase 1) and CYBB (NOX2). In a clinical study based on 182 COVID-19 cases by Violi et al. (2020) [29], the results showed that NOX2 was highly activated in COVID-19 patients. In particular, the degree of NOX2 activation was higher in those with severe conditions. Severe illness and thrombosis in COVID-19 patients were closely related to oxidative stress caused by NOX2 activation. A study involving 453 patients with severe COVID-19 suggested that FCGR2A may be associated with the risk of death in patients infected with SARS-CoV-2 [30]. The deficiency in IRF9 is associated with impaired control of multiple viruses (including SARS-CoV-2), building the functional relationship between IRF9 and respiratory viral infection [31]. OAS1 (2′–5′-oligoadenylate synthetase 1) is regarded as a SARS-CoV-2 restriction factor capable of initiating efficient blockade of viral replication [32].

Based on the result of molecular function identification (Figure 3C), RAGE receptor binding was the most significant function carried out by the identified DEGs. Studies have confirmed that members of the renin–angiotensin system (RAS) played a decisive pathogenic role in mediating organ damage caused by SARS-CoV-2 infection. RAGE is a member of the immunoglobulin superfamily and plays an important role in lung pathological states, including ARDS and pulmonary fibrosis. RAGE overexpression or hyperactivation is critical for the deleterious effects of RAS in multiple major comorbidities caused by SARS-CoV-2 infection, including hypertension, diabetes and cardiovascular disease. RAGE could be considered as a potential therapeutic target in patients with COVID-19 to improve multi-organ pathology caused by the virus [33].

In the PPI network established based on the DEGs, hub genes were identified based on the topological metric (i.e., degree), and these hubs could be key therapeutic targets or molecular markers in COVID-19 patients with cancer and associated with various pathological and biological mechanisms. According to their degree values, a total of five hub genes were selected (Figure 4B), including TLR4, STAT1, IRF1, STAT2 and CYBB. In this network, the gene with the largest degree value was TLR4, which was one of the most extensively studied toll-like receptor (TLR) family members. In different solid tumor types, TLR4 is used as a biomarker to reveal tumor proliferation, differentiation, metastasis, prognosis and patient survival status [34]. In addition, the overactivation of TLR4 leads to prolonged or excessive innate immune responses after infection with SARS-CoV-2. Therefore, SARS-CoV-2-induced myocarditis, ARDS and multiple-organ damage may be due to TLR4 activation, aberrant TLR4 signaling and excessive inflammation in COVID-19 patients [35].

Sorted by adjusted p-value, the top five candidate drug molecules predicted from the 22 common DEGs were Tamibarotene CTD 00002527, suloctidil HL60 UP, Phorbol 12–myristate 13–acetate CTD 00006852, acetohexamide PC3 UP and 3′-Azido–3′-deoxythymidine CTD 00007047 (Figure 6). Tamibarotene was the potential drug molecule with the smallest adjusted p-value. Based on the findings of Liao et al. (2021), they clarified that tamibarotene is a retinoid derivative with broad-spectrum antiviral activity. In a hamster model, intranasal delivery of tamibarotene by spray freeze drying (SFD) technique showed efficacy against SARS-CoV-2 virus [36]. Tamibarotene has shown efficacy in inhibiting tumor cell growth in advanced hepatocellular carcinoma patients with acceptable tolerability [37]. Similarly, tamibarotene may lead to a new strategy for the treatment of acute promyelocytic leukemia (APL) through improved relapse-free survival [38].

IFITM2 and TNFSF10 were the two DEGs that overlapped when comparing the gene expression levels from patients of COVID-19, cancer, ARDs and PF. IFITM2 was upregulated in COVID-19 patients without cancer, which can be supported by the conclusion from previous study that IFITM2 inhibited the entry of SARS-CoV-2 virus [39]. However, compared with recovered COVID-19 patients without cancer, IFITM2 was downregulated in COVID-19 patients with hematologic cancer (exitus) (Figure 10K). Thus, it may impair the inhibition of SARS-CoV-2 entry, making the patients more prone to developing severe clinical consequences. In addition, IFITM2 also has the effect of promoting cancer. Evidence showed that IFITM2 was highly expressed in gastric cancer and renal clear cell carcinoma, which associates with poor survival [40,41].

Another study indicated that TNFSF10 (TRAIL) was upregulated in bronchoalveolar lavage fluid (BALF) and PBMC of patients infected with SARS-CoV-2 [42]. We also found that TNFSF10 as an immune-related gene was upregulated in COVID-19 patients with cancer (Figure 2F and Figure 10K). Additionally, Han et al. (2022) revealed that TNFSF10 plays an important role in the regulation of antiviral immune responses in triple-negative breast cancer [43].

N^6^–methyladenosine (m6A) has been identified to be a frequent RNA modification that affects transcript functions in virus-infected cells. SARS-CoV-2 RNA is modified by m6A [44]. Further, Li et al. (2021) revealed the mechanism of m6A modification in evading the host immune responses to viral infection [45]: RIG–I binds to SARS-CoV-2 viral RNAs thereby inhibiting their activity. In host cells, m6A regulator METTL3 adds m6A modification to SARS-CoV-2 RNA, which reduces the binding of viral RNA to RIG–I, thereby inhibiting the sensing and activation of the innate immune response. We explored how METTL3 affects downstream molecules at the transcriptional level after SARS-CoV-2 infection. The GSE167075 dataset contains the transcriptome data after depletion of the m6A methyltransferase METTL3 in host cell infected with SARS-CoV-2. Then the integrated analysis revealed 1 common gene is shared among 32 common DEGs (COVID-19 vs. control) and the DEGs obtained by the GSE167075 dataset (Figure S2A). Compared with healthy control, the common gene SERPINA1 was upregulated in both GSE164571 (Figure S2B) and GSE147507 (Figure S2C) datasets. In the volcano plot (Figure S2D), 451 upregulated and 940 downregulated genes were included in the obtained 1391 DEGs from the GSE167075 dataset. Compared with controls, SERPINA1 was downregulated after METTL3 depletion, which illustrates the therapeutic potential of targeting m6A pathways (e.g., METTL3/SERPINA1 axis) to restrict SARS-CoV-2 reproduction.

NK cells are an important part of the innate immune system, which can suppress tumor cells in humans through a variety of mechanisms [46]. NK cells are also the first line of defense against invading viruses because of their ability to target infected cells directly without the need for specific antigen presentation [47]. Figure 10B showed that IFITM2 was highly expressed in NK cells of COVID-19 patients without cancer. How IFITM2 regulate immune responses through NK cells is unknown in SARS-CoV-2-infected cancer patients. This requires more clinical samples and experimental data to be explored in the future.

The two main types of blood monocytes are CD14++/CD16- and CD14+/CD16+ monocytes. Among them, CD14+/CD16+ monocytes are regarded as proinflammatory. CD14+/ CD16+ monocytes are key players in infection and inflammation [48]. In addition, CD14+/CD16+ monocytes are involved in antitumor response [49]. Excessive monocyte activation is accompanied by cytokine storm and subsequent acute lung injury, leading to ARDS, which is a direct consequence of infection with SARS-CoV-2 [50]. Figure 10E,F and Figure 10I,J indicated that both TNFSF10 and IFITM2 were highly expressed in CD14+ and CD16+ monocytes of COVID-19 patients without cancer. Changes in the expression levels of these two potential biomarkers may affect the immune response of COVID-19 patients, and they could also be helpful guiding the prognosis and treatment of SARS-CoV-2-infected cancer patients with further evaluation.

## 4. Materials and Methods

### 4.1. GEO Dataset Used in This Study

We collected five datasets from GEO profiles (https://www.ncbi.nlm.nih.gov/geoprofiles/ (accessed on 20 December 2021)), including GSE164571, GSE147507, GSE89953, GSE40839 and GSE153610. To determine the genetic interrelationships shared among COVID-19 patients without and with cancer, ARDS and PF, the corresponding GEO datasets were used. The GSE164571 dataset contains NanoString data from 12 PBMC samples [12], where the samples were divided into 3 groups: five COVID-19 patients without cancer, five COVID-19 patients with cancer and two healthy controls. Table 2 describes the details of the patients and healthy controls from GSE164571. The GSE147507 dataset depicts transcriptional response to SARS-CoV-2 infection [13]. The GSE89953 dataset reveals transcriptional changes in AM and PBM from patients with ARDS, and 26 paired AM and PBM samples were used for RNA-seq analysis in this study. For the PF dataset (GSE40839), there are 10 normal human lung tissues and 8 scleroderma-associated fibrotic human lung tissue samples.

### 4.2. Identification of Common DEGs among COVID-19 without and with Cancer, ARDS and PF

A critical step in this study is to acquire the DEGs of datasets GSE164571, GSE147507, GSE40839 and GSE89953. The DEGs identification based on the GSE164571 dataset was done by GEOquery [51] and limma package in GEO2R tool (https://www.ncbi.nlm.nih.gov/geo/geo2r/ (accessed on 20 December 2021)). The Benjamini-Hochberg method was applied to adjust the *p*-values [52]. The identification of the common DEGs from GSE147507, GSE40839 and GSE89953 datasets was performed through the use of the R programming language. Adjusted *p*-value < 0.05 and |log2FC| ≥ 1 were used as cutoff criteria to obtain significant DEGs from all the datasets. The common DEGs were identified through the overlap from a Venn diagram online tool called jvenn [53].

For the analysis of GSE40839 and GSE89953 datasets, the RNA-seq data were normalized and then visualized using PCA and UMAP, which were plotted using R packages, including ggplot2 [54] and umap.

### 4.3. Acquisition and Classification of Immune-Related Genes

A complete list of immune-related genes was obtained from the ImmPort website (https://www.immport.org/shared/genelists (accessed on 20 December 2021)) [55] and was used to identify the DEGs that were related to the immune system. The classification of the immune-related DEGs was performed and visualized by R package ggalluvial [56].

### 4.4. Gene Ontology (GO) and Pathways Analysis Based on Common DEGs

Gene set enrichment is a statistical-based computational method used to determine whether a pre-defined gene set shows statistically concordant differences, such as their functions, related pathways, under different biological conditions [57]. GO terms based on the identified common DEGs are obtained from the Enrichr (https://maayanlab.cloud/Enrichr/ (accessed on 20 December 2021)) platform [58], and the GO terms are divided into three categories including biological process, cellular component and molecular function [59]. KEGG [60], WikiPathways [61] and Reactome [62] databases were used to analyze pathways involved by identified common DEGs. Pathway-related results were also implemented using the Enrichr platform.

### 4.5. Analysis of PPI Network and Hub Genes Identification

The identification of protein–protein interaction sites can provide prominent insights into protein functions, which is an important step in systems biology and drug development [63]. We used the STRING database (https://string-db.org/ (accessed on 20 December 2021)) [64] to construct the PPI network based on shared DEGs to delineate the functional and physical interactions between proteins. For a better visualization of the network, the obtained PPI network was further analyzed and modified using Cytoscape (https://cytoscape.org/ (accessed on 20 December 2021)) [65]. There are many nodes and edges in the PPI network, where the nodes represented the proteins/genes and the links depicted their interactions. The nodes with the most interactions were regarded as hub genes. The degree algorithm is applied to extract hub genes in the PPI network.

### 4.6. Analysis of TF–DEG Interactions and miRNA–DEG–TF Coregulatory Network

TFs regulate transcription by recognizing specific DNA sequences [66]. The NetworkAnalyst (https://www.networkanalyst.ca/ (accessed on 20 December 2021)) platform [67] was used to perform the analysis identifying TF-gene interactions related to the common DEGs. Transcription factors and gene targets were based on ChIP-seq data sourced from the ENCODE database (https://www.encodeproject.org/ (accessed on 20 December 2021)) [68] included in the NetworkAnalyst platform. TF–miRNA coregulatory interactions were obtained from the RegNetwork repository (http://www.regnetworkweb.org (accessed on 20 December 2021)) [69]. And then the network showing miRNAs and regulatory TFs that regulate DEGs of interest were also visualized by the NetworkAnalyst platform.

### 4.7. Prediction of Drug Candidate Molecules

To predict the possible drug candidates targeting the COVID-19 patients with cancer, identified common DEGs were provided as an input in DSigDB [70] in the Enrichr platform. Based on drug-protein interactions, top 5 drug candidates were predicted and the details of these drugs were obtained from the DrugBank database (http://www.drugbank.ca/ (accessed on 20 December 2021)) [14].

### 4.8. Visualization of Single-Cell RNA-Seq Data

Specific single-cell RNA-seq datasets were visualized by the UCSC Cell Browser (https://cells.ucsc.edu (accessed on 20 December 2021)) [71]. In the UMAP plot, canonical cell markers were used to annotate different clusters by cell identity. The expression levels of identified specific genes were distinguished based on their corresponding frequencies in different clusters. Sample acquisition and all parameter settings for single-cell RNA-seq analysis were described in the publications of Zhang et al. (2020) [25] and Stephenson et al. (2021) [26].

## 5. Conclusions

In this study, we compared the gene expression patterns between COVID-19, ARDS and PF in cancer patients infected with SARS-CoV-2 and identified common DEGs among them with more detailed analysis of these DEGs. TF–DEG interactions and miRNA–DEG–TF coregulatory networks demonstrate the way in which DEGs expression is regulated by changes at the transcriptional and post-transcriptional levels. Based on the principle of drug–protein interaction, the top drug candidate molecule, Tamibarotene, exhibits antiviral and anticancer properties, and therefore might be helpful for effective therapeutic targets in COVID-19 patients with cancer. In addition, the common DEGs among ARDS, PF and COVID-19 patients with and without cancer are TNFSF10 and IFITM2. These two genes may be regarded as potential therapeutic targets in the treatment of COVID-19 patients with cancer. Moreover, the immune responses were activated after SARS-CoV-2 infection in COVID-19 patient with or without cancer. Targeting TNFSF10 and IFITM2 in CD14+ and CD16+ monocytes may affect the immune response of COVID-19 patients with cancer. Specifically, changes in the expression level of TNFSF10 in monocytes can be considered as an immune signature in COVID-19 patients with hematologic cancer.

## Figures and Tables

**Table 1:** Suggested TOP 5 drug compounds.

No.	Name of Drugs	DrugBank Accession Number [14]	Chemical Formula	Chemical Structure	Background
1	Tamibarotene	DB04942	C_22_H_25_NO_3_	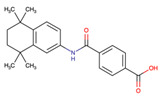	Tamibarotene is a novel synthetic retinoid for acute promyelocytic leukaemia (APL) [15]. Tamibarotene is currently approved in Japan for treatment of recurrent APL and is undergoing clinical trials in the United States [16].
2	Suloctidil	DB13340	C_20_H_35_NOS	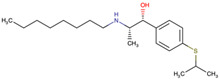	A peripheral vasodilator that was formerly used in the management of peripheral and cerebral vascular disorders [17].
3	Phorbol 12–myristate 13–acetate	/	C_36_H_56_O_8_	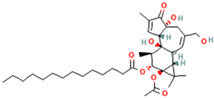	It has a role as a protein kinase C agonist, an antineoplastic agent, a reactive oxygen species generator, a plant metabolite, a mitogen, a carcinogenic agent and an apoptosis inducer [18,19,20].
4	Acetohexamide	DB00414	C_15_H_20_N_2_O_4_S	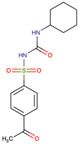	A sulfonylurea hypoglycemic agent that is metabolized in the liver to 1-hydrohexamide [21].
5	3′-Azido–3′-deoxythymidine	DB00495	C_10_H_13_N_5_O_4_	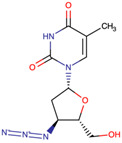	A dideoxynucleoside compound in which the 3’-hydroxy group on the sugar moiety has been replaced by an azido group. This modification prevents the formation of phosphodiester linkages which are needed for the completion of nucleic acid chains. The compound is a potent inhibitor of HIV replication, acting as a chain-terminator of viral DNA during reverse transcription. It improves immunologic function, partially reverses the HIV-induced neurological dysfunction and improves certain other clinical abnormalities associated with AIDS. Its principal toxic effect is dose-dependent suppression of bone marrow, resulting in anemia and leukopenia [22].

**Table 2:** The details of the patients and healthy Controls from the GSE164571 dataset.

Group	Serial Number	Sex	Age	Neoplastic Disease	Degree of Severity	Sample Source	Anticancer Treatment	Days between COVID-19 First Positive Swap and Blood Collection
Healthy Donors	HD Y	F	54	N.A.	N.A.	PBMCs	N.A.	N.A.
	HD 7	M	51	N.A.	N.A.	PBMCs	N.A.	N.A.
COVID-19 patients without cancer	Sand-003	M	60	N.A.	Critical	PBMCs	N.A.	37
	Sand-004	F	69	N.A.	Critical	PBMCs	N.A.	57
	Sand-007	F	88	N.A.	Moderate	PBMCs	N.A.	37
	Sand-010	M	65	N.A.	Mild	PBMCs	N.A.	2
	Sand-100	M	68	N.A.	Severe	PBMCs	N.A.	39
COVID-19 patients with cancer	Sand-005	M	69	Clear cell renal cell carcinoma (CCRCC)	Severe	PBMCs	No treatment (neo-diagnosis)	37
	Sand-006	M	74	Chronic Lymphatic Leukemia (CLL)	Critical	PBMCs	No treatment (neo-diagnosis)	42
	Sand-008	M	70	Lung cancer	Severe	PBMCs	No treatment	24
	Sand-009	F	74	Gastrointestinal Cancer	Mild	PBMCs	No treatment (neo-diagnosis)	2
	Sand-011	M	69	Classical mixed cellularity Hodgkin Lymphoma	Severe	PBMCs	No treatment (neo-diagnosis)	55

## Data Availability

Data are contained within the article or supplementary material.

## Supplementary Materials

The supporting information can be downloaded at: www.mdpi.com/article/10.3390/ijms232415698/s1.

## Abbreviations

AM: alveolar macrophages; ARDS: acute respiratory distress syndrome; APL: acute promyelocytic leukemia; BALF: bronchoalveolar lavage fluid; COVID-19: coronavirus disease 2019; DEGs: differentially expressed genes; DSigDB: Drug Signatures Database; GEO: Gene Expression Omnibus; KEGG: Kyoto Encyclopedia of Genes and Genomes; m6A: N^6^–methyladenosine; NK: Natural-killer; RAS: renin–angiotensin system; PBM: peripheral blood monocytes; PBMCs: peripheral blood mononuclear cells; RAGE: receptor for advanced glycation end products; PF: Pulmonary fibrosis; PPI: protein–protein Interaction; SARS-CoV-2: severe acute respiratory syndrome coronavirus 2; SFD: spray freeze drying; TF: transcription factor; TLRs: Toll-like receptors.
