# Supplementary material for: Integrated Analysis of Bulk RNA-Seq and Single-Cell RNA-Seq Unravels the Influences of SARS-CoV-2 Infections to Cancer Patients
Source: Int J Mol Sci. 2022 Dec 10;23(24):15698. doi: 10.3390/ijms232415698 (PMC9779348; doi:10.3390/ijms232415698)
Supplement: Supplementary file 1 [file ijms-23-15698-s001.zip › Supplementary methods and figure legend.pdf]

1. Supplementary Methods

1.1 Identification of differentially expressed genes (DEGs) and common gene identification between SARS-CoV-2 and m^{6}A modification (METTL3)

The identification of DEGs from GSE167075 dataset (https://www.ncbi.nlm.nih.gov/geo/query/acc.cgi?acc=GSE167075) [1] was performed through using the R programming language. Adjusted P-value < 0.05 and $|\log_2 FC| \geq 1$ was regarded as cutoff criteria to obtain significant DEGs from all the datasets. The common genes among GSE164571, GSE147507 and GSE167075 datasets were identified through a Venn diagram online tool called jvenn [2].

1.2 Acquisition and classification of immune-related genes

A list of immune-related genes was obtained from the ImmPort website (https://www.immport.org/shared/genelists) [3]. Classification of immune-related genes was visualized by R package ggalluvial [4].

2. Supplementary figure legends

Figure S1.The related pathways of coronavirus disease (COVID-19) from KEGG. Enriched genes from common DEGs were marked by red circles.

Figure S2. Identification of DEGs and screening of common DEGs between SARS-CoV-2 and m^{6}A modification (METTL3). (A)The Venn diagram showed the common DEGs among GSE164571, GSE147507 and GSE167075 datasets. The volcano plot depicted the DEGs between COVID-19 patients without cancer and healthy controls by using the GSE164571 (B) and GSE147507 (C) datasets (Adjust P-value<0.05, $|\log_2 FC| \geq 1$). (D) The volcano plot showed the DEGs between METTL3 depletion and

control by using GSE167075 dataset (Adjust P-value<0.05, $|log_2FC|\geq1$). The common gene SERPINA1 was marked in the volcano plots.

3. Supplementary table legends

Table S1.The details of the patients from GSE153610 dataset.

Supplementary references

1. Li N, Hui H, Bray B, et al. METTL3 regulates viral m6A RNA modification and host cell innate immune responses during SARS-CoV-2 infection. *Cell Rep*. May 11 2021;35(6):109091. doi:10.1016/j.celrep.2021.109091
2. Bardou P, Mariette J, Escudie F, Djemiel C, Klopp C. jvenn: an interactive Venn diagram viewer. *BMC Bioinformatics*. Aug 29 2014;15:293. doi:10.1186/1471-2105-15-293
3. Bhattacharya S, Andorf S, Gomes L, et al. ImmPort: disseminating data to the public for the future of immunology. *Immunol Res*. May 2014;58(2-3):234-9. doi:10.1007/s12026-014-8516-1
4. Brunson J. ggalluvial: Layered Grammar for Alluvial Plots. *Journal of Open Source Software*. 2020;5(49)doi:10.21105/joss.02017
