# Supplementary figures and images for: Integrated Analysis of Bulk RNA-Seq and Single-Cell RNA-Seq Unravels the Influences of SARS-CoV-2 Infections to Cancer Patients
Source: Int J Mol Sci. 2022 Dec 10;23(24):15698. doi: 10.3390/ijms232415698 (PMC9779348; doi:10.3390/ijms232415698)

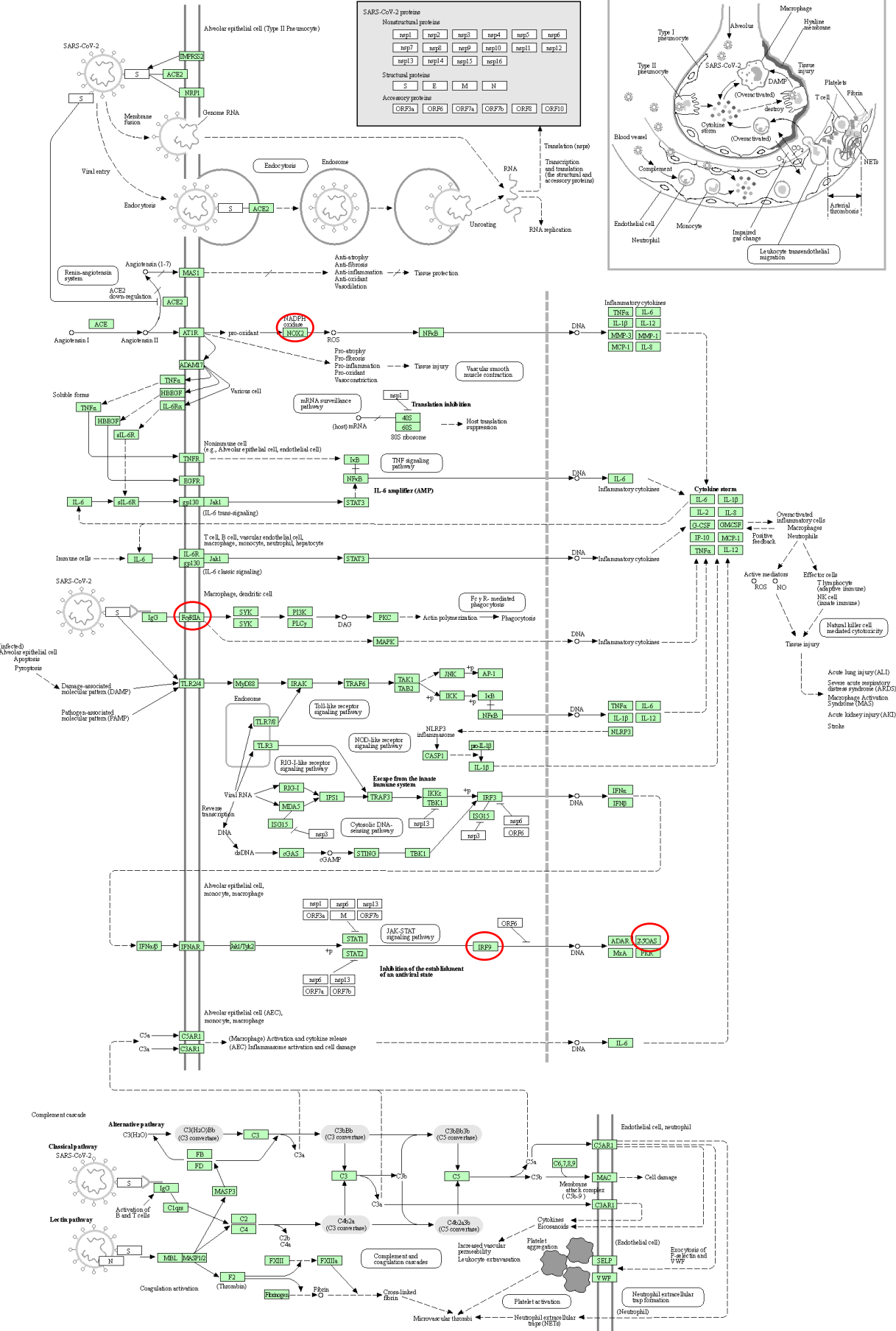

Supplement: Supplementary file 1 [file ijms-23-15698-s001.zip › Figure S1.pdf]

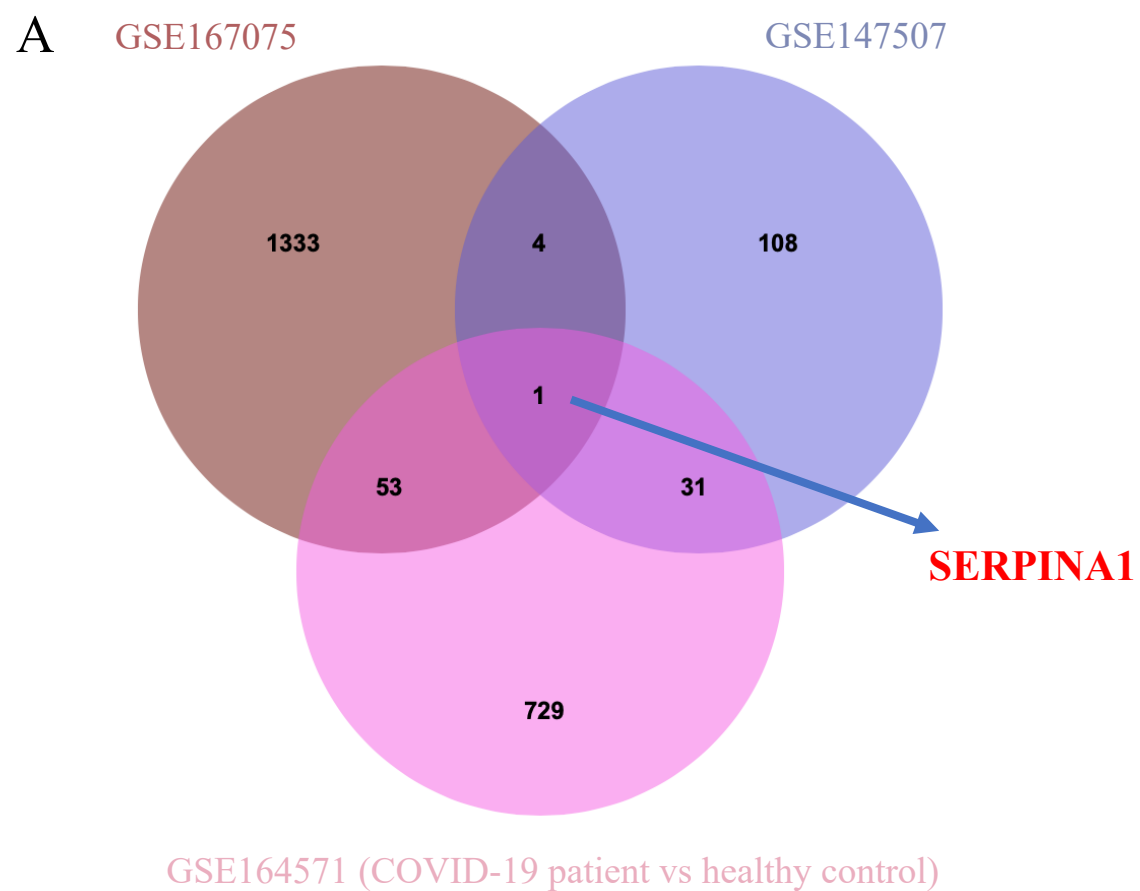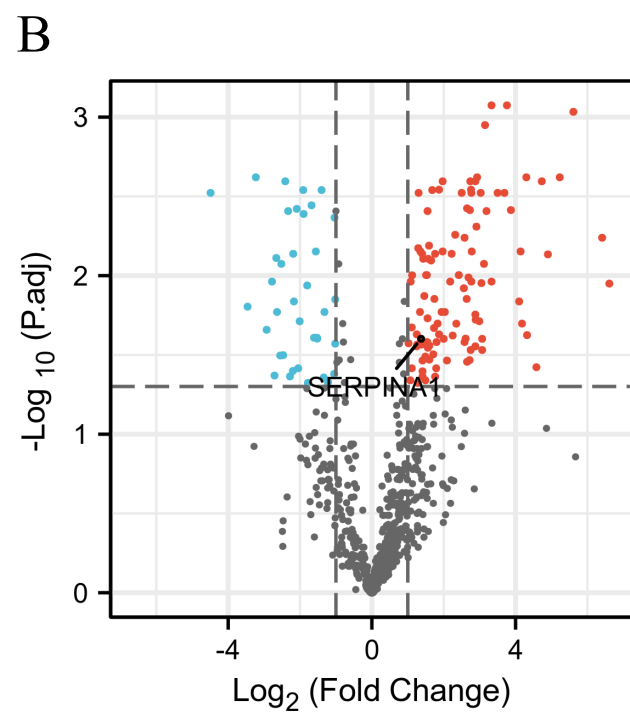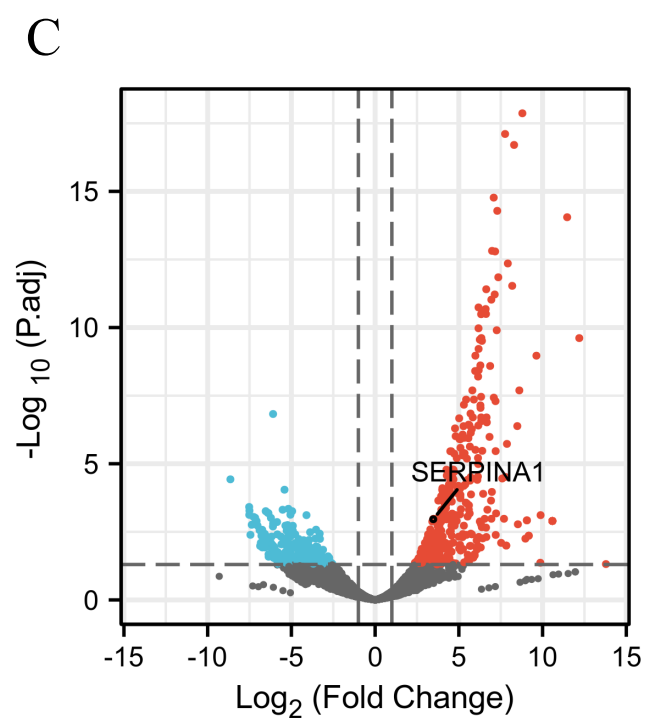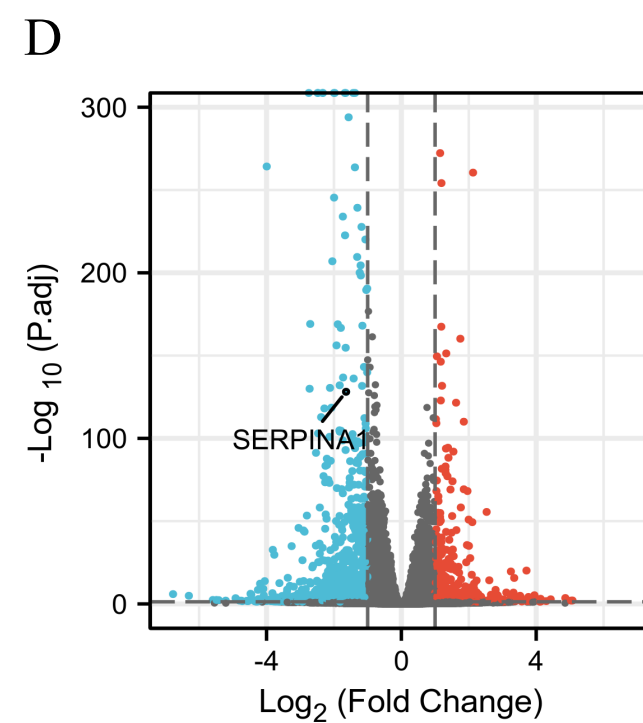

Supplement: Supplementary file 1 [file ijms-23-15698-s001.zip › Figure S2.pdf]
